# On Filing the Teeth

**Published:** 1845-12

**Authors:** J. Robinson

**Affiliations:** Dentist to the Metropolitan Free Hospital.


### ARTICLE IX.

*On Filing the Teeth.* By J. ROBINSON, ESQ., *Dentist to the Metropolitan Free Hospital.*

THE operation of filing the teeth is one in which the practical dentist is daily called upon to exercise his ingenuity and skill, and one which, if effectively and judiciously performed at the commencement of the disease, will be, in most instances, attended with beneficial results. The teeth that are most generally attacked with caries, and for which the application of the file is more frequently brought into request, are the four central incisors and canines of the upper jaw, although, in many instances, it may be used with success to the bicuspides and molares of both jaws. The permanent central and lateral incisores of the upper jaw, frequently decay at an early period at their sides. This arises either from a too crowded state of the mouth, and the undue influence exercised on the parts by their too rapid advance before the maxillary arch is sufficiently developed to admit the increased size; or from the patient at that period neglecting to perform those daily ablutions so essential and necessary to the health of these organs. In either case it unquestionably forms the exciting cause of caries in those situations, which if allowed to extend beyond a certain point, renders the operation both difficult and dangerous to the tooth itself, owing to the confined space the operator has to use his instruments with that force so requisite to the well packing of the gold to the exclusion of all foreign substances, without the liability of fracturing the enamel; this difficulty must have been experienced by all practical dentists, and more particularly when the disease has extended to the cutting edge of the tooth, from the impracticability of the tooth of forming a cavity of a proper and sufficient form for retaining the metal. For even should this be effected to the satisfaction of the operator, still the chances are that the tooth will be fractured in an attempt to stop it, or if this misfortune does not occur, the stopping in these situations generally becomes loose a few months afterwards. Hence arises the necessity of filing in the early stages of caries in preference to stopping. In every case which requires the use of the file, the dentist, in my opinion, ought not to be content with merely dividing the teeth, but should extend the operation until the whole disease of the tooth is eradicated and presents a surface as white as the other portion of the tooth which is in a healthy condition. For it should be remembered that a considerable portion of a tooth can be filed away without the slightest injury, if the operation be performed with caution, and the posterior portion removed without any perceptible disfigurement; and, in many cases, the caries can be removed by scraping away with an instrument without having recourse to the file. Young persons are more liable to experience pain in the operation of filing the teeth than adults, owing to the parts being more highly organized and more susceptible of being excited. In any case when the introduction of the file is followed by pain the operation ought to be deferred for a few days, and treated as I shall hereafter mention. Although I am no advocate for the removal of the natural covering of the teeth (enamel) if it can be avoided, still necessity frequently compels the practitioner to have recourse to it for preserving those valuable adjuncts to personal beauty for many years.

The cases in which I have found the file to be attended with success, are those of the four front incisors, the canines, and bicuspides, and frequently, if the position be favorable, the molares; in these, however, success is not so certain. Owing to their presenting such large surfaces the disease generally extends too deep to be removed by the file; it is, however, judicious to make the attempt, and if not successful, they can be preserved by stopping. The manner in which I proceed to remove incipient caries from between the two centrals or laterals, so as to cause as little disfigurement to the anterior parts as possible, is this: I first make a clear division to the gum with a moderately rough dividing file; I then remove the caries from the posterior part of the tooth with a bent file, during which operation the teeth are supported by the finger and thumb of the other hand to steady them; after having eradicated the caries, I make use of a file much finer in the texture than the former for the purpose of removing the roughness wherein any foreign substance may lodge. I afterwards make use of a third file still finer in the teeth, and lastly employ the end of a piece of common cane with a little chalk and finely-powdered pumice stone, with which I polish the surface. If during the operation the patient experience much pain, I do not proceed for a few days until the irritation has subsided; in the meantime, the constant application of spirit of camphor and morphiæ will materially assist in allaying it. After the operation has been finished, I provide my patients with a similar piece of cane cut thin, which I order to be used night and morning in the same way, for the purpose of keeping up the polish and removing any accumulation that might possibly have collected.

Notwithstanding, however, all these precautionary measures I have alluded to, it does not follow that caries should not again attack a part once weakened by disease, but more generally some other part of the tooth becomes affected, which must be attributed to some general defect in its structure. I have frequently, after dividing a tooth, discovered near its cutting edge a large cavity, which it would be impossible to remove without destroying more than half the tooth and disfiguring the patient; in any attempt to stop it with gold the chances would be either a fracture or an imperfect stopping. I have in these cases substituted gum mastich steeped in warm water, an admirable substitute which has remained in the cavity for months, and can be renewed at pleasure by the patient. In many instances I have examined the teeth three and four years afterwards, and have found the cavities perfectly healthy and not in the least indicating a return of the disease.—*London Forceps.*

